# Music Score Recognition Method Based on Deep Learning

**DOI:** 10.1155/2022/3022767

**Published:** 2022-07-07

**Authors:** Qin Lin

**Affiliations:** Art College of Guizhou University of Finance and Economics, Guiyang 550001, Guizhou, China

## Abstract

In recent years, the recommendation application of artificial intelligence and deep music has gradually become a research hotspot. As a complex machine learning algorithm, deep learning can extract features with value laws through training samples. The rise of deep learning network will promote the development of artificial intelligence and also provide a new idea for music score recognition. In this paper, the improved deep learning algorithm is applied to the research of music score recognition. Based on the traditional neural network, the attention weight value improved convolutional neural network (CNN) and high execution efficiency deep belief network (DBN) are introduced to realize the feature extraction and intelligent recognition of music score. Taking the feature vector set extracted by CNN-DBN as input set, a feature learning algorithm based on CNN&DBN was established to extract music score. Experiments show that the proposed model in a variety of different types of polyphony music recognition showed more accurate recognition and good performance; the recognition rate of the improved algorithm applied to the soundtrack identification is as high as 98.4%, which is significantly better than those of other classic algorithms, proving that CNN&DBN can achieve better effect in music information retrieval. It provides data support for constructing knowledge graph in music field and indicates that deep learning has great research value in music retrieval field.

## 1. Introduction

With the rapid development of the Internet and information society, intelligent, globalized, and nongroup information features widely exist in our life. The information age makes us transfer resources in a more convenient way. In the era of big data, with the development of data mining technology, the technology of mining and refining massive data and converting it into valuable information has gradually become popular and mature. People get a lot of video, audio, and text information through the computer network and get spiritual enjoyment from it, among which music is very important for human beings [1]. With the development of the Internet era, people require the retrieval of music information to be more rapid, accurate, convenient, and efficient. However, music has many characteristics of its own, such as tone, melody, timbre, and tone strength, but the characteristics of music itself are difficult to be embodied, unlike traditional texts that can be described in an intuitive way. With the development of digital media economy, music information retrieval and recognition has become a hot research field for many scholars. With the emergence of deep learning, music classification technology has entered a new development period [2, 3]. Deep learning in areas such as image processing and speech recognition has been more widely used than the traditional machine learning method, and the scholars have also started to use deep learning technology research in the field of music information retrieval related issues. Not only is the label of musical instrument of great significance to the classification of music type, but also it can be used to predict the emotion and music scene contained in music, so the identification and classification of musical instrument also play an important role in the field of music information retrieval. If we know the instrument that plays a particular piece of music, then we can optimize the automatic classification of music according to the characteristics of the instrument used [4]. Because the research on musical instrument recognition and classification can help people to study the recognition and classification of other music information retrieval fields, the research on musical instrument recognition and classification has attracted more and more attention of scholars recently.

The proposal and application of the concept of deep learning is the biggest breakthrough in the 21st century. In 2011, Microsoft used deep neural network (DNN) for the first time, which has achieved remarkable results in speech recognition tasks. DNN is attracting more and more attention in speech recognition. Deep learning (DL) is the development of artificial neural network, which has a good application effect in the fields of speech and image and is currently considered a research hotspot. With the development of the times, the amount of network multimedia audio is increasing day by day. How to effectively retrieve the music information that people need has become an urgent problem to be dealt with by music information retrieval technology. Before the emergence of deep learning technology, the research on music classification mainly focused on the selection of feature sets and classifiers [5]. With the emergence of open music datasets, there are more and more research works in the field of music classification. However, most of the research works are still limited to the methods that take the features of the bottom of audio as feature sets and traditional machine learning algorithms as classifiers. The correlation between audio features and music categories is elusive, and the effect of classification using these underlying features is not stable, which largely depends on the selected feature set, and it is difficult to deal with large-scale music data using traditional classification algorithms. The mentioned problems limit the development of music classification research. With the rise of deep learning, classification methods based on deep neural network begin to emerge [6, 7]. Musical instrument recognition is an important field of audio retrieval, which involves not only the acoustic properties of sound source but also the perception psychology of human ear.

Based on previous studies, on the basis of considering the deep learning strong ability of feature extraction in the field of image recognition, this paper explores the innovative deep learning algorithms in the research and application of the background music classification recognition; on the basis of the classical neural network, the eigenvalue vector was introduced; first of all is the preprocessing of sample datasets combined with characteristic parameters change, followed by strengthening the characteristic value; then, the classification model of music score after deep supervised learning is established, and the model application comparison test is carried out on the test samples. The experiment proves that the recognition rate of the improved algorithm in music recognition is as high as 98.4%, which is obviously better than those of other classical algorithms. The main innovation of this paper is that the proposed model shows more accurate recognition ability and good execution performance in recognition of multiple types of polyphony music.

## 2. Basic Theory of Deep Learning

Deep learning itself is a kind of artificial neural network, on which many improvements have been made to deepen the depth and improve the complexity of the network. It can be said that deep learning is a series of related technologies generated for better application of deeper and more complex networks [8]. Neurons in deep learning models mimic the way neurons work in the brain, trying to mimic the way stimuli communicate with neurons in the brain. Since the development of deep learning, many classical network structures have been born, and they have achieved achievements that traditional machine learning could not achieve. Convolutional neural network adopts the structure pattern of human neural tissue, sets up the convolution layer, and provides the associated subsampling layer for it. It uses certain rules to connect the upper and lower layers and the adjacent neural tissues to establish a forced and local association relationship in each layer. These units have a continuous receiving domain in space.

**Figure 1:**
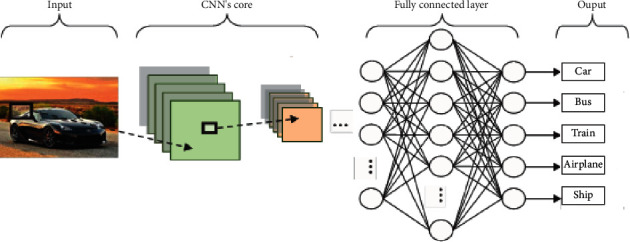
Typical structure of CNN [10].

Convolutional neural network has the characteristics of reducing network parameters and learning local features. Compared with traditional fully connected neural network, the biggest difference is that only a part of nodes are connected between adjacent layers, so as to avoid the problem of too many parameters in the fully connected layer. By reducing the parameters, the convolutional neural network can calculate faster and avoid the overfitting problem [9]. The typical structure of CNN is shown in Figure 1. Figure 1 is reproduced from the work of Chen and Tang (2021) [10] (under the Creative Commons (Attribution License/public domain)).

### 2.1. Input Layer

Consider the input of a channel number of data *F*_0_; the standard image processing *F*_0_=3, exactly corresponding to the three color channels of the computer: red, green, and blue [11]. Set the input after processing as $\tilde{X}$, and the specific calculation process can be expressed as

(1) $$\begin{array}{c} {\tilde{X}}_{f,j,k}^{\left(i\right)}=\frac{X_{f,j,k}^{\left(i\right)}-{\overset{⌢}{X}}_{f,j,k}^{\left(i\right)}}{\sigma {}_{f,j,k}}, \end{array}$$

where ${\overset{⌢}{X}}_{f,j,k}^{\left(i\right)}=1/l\sum_{i=0}^{l-1}X_{f,j,k}^{\left(i\right)}$ and $\sigma_{f,j,k}=\sqrt{1/l\sum_{i=0}^{l-1}{\left(X_{f,j,k}^{\left(i\right)}-{\overset{⌢}{X}}_{f,j,k}^{\left(i\right)}\right)}^{2}}$.

### 2.2. Padding

**Figure 2:**
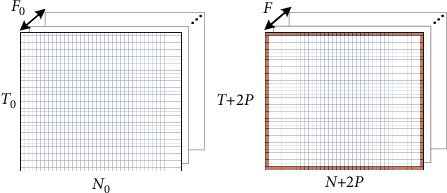
Padding of the feature map.

The completion operation of size is *P*, 1 is added to the beginning and end of each row and column of the feature graph [12]. The padding of the feature map is shown in Figure 2; the red part represents the part of completion, where *P*=1.

### 2.3. Convolution

**Figure 3:**
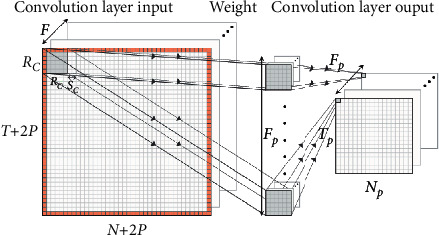
Convolution operation diagram [10].

The convolution layer is the core network layer in the convolutional neural network. It mainly carries out the convolution operation, extracts local features based on the spatial local correlation of input data, and forms the overall features by connecting these local features. CNN is named from the unique convolution operation in the model, which is also the most important part of the model.  Figure 3 illustrates the convolution operation diagram.  Figure 3 is reproduced from the work of Chen and Tang (2021) [10] (under the Creative Commons (Attribution License/public domain)). As can be seen from the figure, neurons at each adjacent level are compulsively connected with each other and have correlation relations. The input filtering function of this structure in local space has strong adaptability. It mainly includes data input layer and hidden layer, and each hidden layer is subdivided into convolution layer, sampling layer, and pooling layer. The core of the algorithm is convolution and pooling. The supervised learning mode is used to construct the training network, which mainly includes the preceding training propagation and reverse training propagation. The forward training propagation changes the input training sample matrix in layers, and the output of each layer is the input of the next layer, and finally the convolutional feature matrix is extracted [13, 14].

Set the width of the input data as *N* and the height as *T*; then the width and height of the output image can be calculated by the following formula:

(2) $$\begin{array}{c} N_{p}=\frac{N+2P-R_{c}}{S_{c}}+1,T_{p}=\frac{T+2P-R_{c}}{S_{c}}+1. \end{array}$$

Then the operation can be expressed as

(3) $$\begin{array}{c} a_{f,l,m}^{\left(i\right)}=\sum_{f^{\prime }=0}^{F_{v}-1}\sum_{j=0}^{R_{c}-1}\sum_{k=0}^{R_{c}-1}w_{f^{\prime },j,k}^{\left(v\right)f}h_{f^{\prime },s_{c}l+j,s_{c}m+k}^{\left(i\right)\left(v\right)}, \end{array}$$

where *v* ∈ [0, *N* − 1], *f* ∈ [0, *F*_*v*+1_ − 1], *l* ∈ [0, *N*_*v*+1_ − 1], and *m* ∈ [0, *T*_*v*+1_ − 1]. After each hidden layer, an activation function *g* is required to introduce nonlinear features. After counting completion operation, the input of the next layer is *h*_*f*,*l*+*p*,*m*+*p*_^(*i*)(*v*+1)^=*g*(*a*_*f*,*l*,*m*_^(*i*)(*v*)^).

### 2.4. Pooling

**Figure 4:**
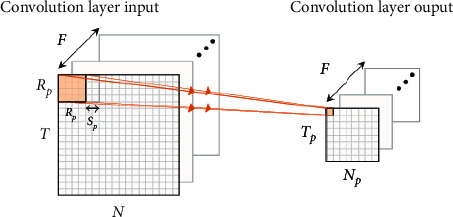
Pooling operation diagram [10].

Figure 4 is the pooling operation diagram. Figure 4 is reproduced from the work of Chen and Tang (2021) [10] [under the Creative Commons [Attribution License/public domain]]. In convolutional neural networks, pooling layer is often used to further adjust the output of the convolutional layer. After extracting object features through the convolutional layer, pooling layer operation usually needs to be inserted between two adjacent convolutional layers. Pooling layer can further reduce the number of neuron connections between convolutional layers, accelerate the learning speed of the model, and alleviate the problem of overfitting. Specifically, the pooling operation computes the local statistical characteristics of a location to replace the elements of the input data on that location region. Through the operation of the pooling layer, the number of parameters in the entire network is reduced, which alleviates the complexity of parameter calculation and storage [15, 16].

The average pooling operation can be expressed as

(4) $$\begin{array}{c} h_{f,l+p,m+p}^{\left(i\right)\left(v+1\right)}=a_{f,l,m}^{\left(i\right)\left(v\right)}=h_{f,s_{p}l+j+p,s_{p}m+k+p}^{\left(i\right)\left(v\right)}k_{\left(f,l,m\right)}^{\left(i\right)\left(p\right)}+p. \end{array}$$

Setting *j*_(*f*, *l*, *m*)_^(*i*)(*p*)^, *k*_(*f*, *l*, *m*)_^(*i*)(*p*)^ represents the pixel index with the largest value in the pooled perception field, and the input of the next layer is got:

(5) $$\begin{array}{c} a_{f,l,m}^{\left(i\right)\left(v\right)}=\sum_{j,k=0}^{R_{p}-1}h_{f,s_{p}l+j+p,s_{p}m+k+p}^{\left(i\right)\left(v\right)}. \end{array}$$

### 2.5. Convolution to the Full Connection Layer

The learning of several convolutional layers and pooling layers can abstract the information in the input data into features with higher information density; that is to say, the convolutional layer and pooling layer can be regarded as the process of data feature extraction and learning [17]. After completing this step, the full connection layer can be used to complete the classification task. This process can be expressed as

(6) $$\begin{array}{c} a_{f}^{\left(i\right)\left(v\right)}=\sum_{f^{\prime }=0}^{F_{v}-1}\sum_{l=0}^{R_{c}-1}\sum_{m=0}^{R_{c}-1}w_{f^{\prime },l,m}^{\left(v\right)f}h_{f^{\prime },l+p,m+p}^{\left(i\right)\left(v\right)}. \end{array}$$

**Figure 5:**
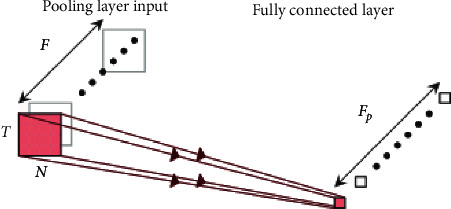
One-dimensional output obtained by convolution [10].

The next level of input is obtained by activating the function *h*_*f*_^(*i*)(*v*+1)^=*g*(*a*_*f*_^(*i*)(*v*)^).  Figure 5 is the one-dimensional output obtained by convolution.  Figure 5 is reproduced from the work of Chen and Tang [10] [under the Creative Commons [Attribution License/public domain]].

### 2.6. Fully Connected Layer

The maximum pooling operation uses the maximum value of the local region as the output result, which can significantly reduce the offset caused by the parameter error of the convolution layer and retain more local feature information of the input data. Common pooling operations include the average value of adjacent rectangular regions and the weighted average function of the distance from the center pixel.

(7) $$\begin{array}{c} a_{f}^{\left(i\right)\left(v\right)}=\sum_{f^{\prime }=0}^{F_{v}-1}w_{f^{\prime }}^{\left(v\right)f}h_{f^{\prime }}^{\left(i\right)\left(v\right)}. \end{array}$$

### 2.7. Output Layer

The last layer of the model is the output layer, outputting

(8) $$\begin{array}{c} a_{f}^{\left(i\right)\left(v\right)}=\sum_{f^{\prime }=0}^{F_{v}-1}w_{f^{\prime }}^{\left(v\right)f}h_{f^{\prime }}^{\left(i\right)\left(v\right)}, \end{array}$$

(9) $$\begin{array}{c} \text{softmax}\left(a_{f}^{\left(i\right)\left(V-1\right)}\right)=\frac{e^{a_{f}^{\left(i\right)\left(V-1\right)}}}{\sum_{f^{\prime }=0}^{F_{v-1}-1}e^{a_{f^{\prime }}^{\left(i\right)\left(V-1\right)}}}, \end{array}$$

where *V* is the total number of layers of the model. *o* is the output function, and the following Softmax [18] function is usually used when the model is used to classify tasks.

After obtaining the features of the training sample through convolution, if the features obtained through convolution are directly used, it is inevitable that there will be a large amount of computation. It is very inconvenient to learn a classifier with extremely high-dimensional feature input, and overfitting phenomenon is very easy to occur in this case. Faced with such a problem, the solution is the feature after convolution, which may be applicable to different regions of the sample. In order to describe the sample with a large dimension, aggregative statistics are a solution for the feature of different locations. People can calculate the average (or maximum) of a particular feature of the sample, and the average (or maximum) is calculated over an area of the sample. This will reduce the dimension, and the overfitting phenomenon will not easily occur. The operation of this aggregation is called pooling. In a different sense, pooling can also be called downsampling.

DL is one of the powerful tools in the current wave of artificial intelligence. Its essence is a rule learning method based on multilayer neural network, which takes massive data as input. The neurons in the input layer receive the input data and transmit the data to the neurons in the hidden layer. The neurons in the hidden layer process the data with activation function and transmit the processed data to the output layer. Finally, neurons in the output layer output the results. In DNN, the more hidden layers, the more complex the structure. At this time, the parameters of the whole network become more, and the abstract representation of each layer is deeper than that of the previous layer, so DNN has more powerful performance. With the booming development of DL, DNN has also been introduced into the field of communication to solve traditional communication problems such as modulation identification. The modulation recognition technology based on DL can give full play to the performance advantages of DNN and make up for the shortcomings of the original algorithms [19].

**Figure 6:**
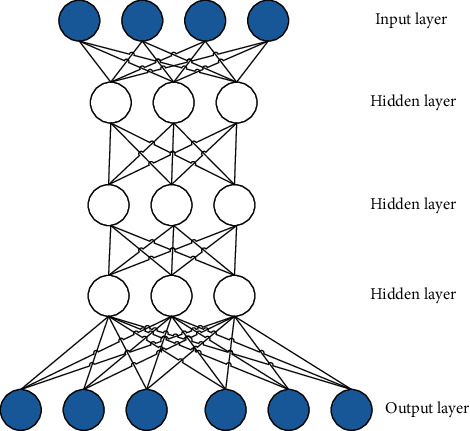
The model structure of CDBM.

The deep confidence network includes hidden layer and visible layer, and the fully connected mode is adopted between layers. The model structure of CDBM is shown in Figure 6.

The restricted Boltzmann mechanism is used in DBN sample model training to form neural network perception, and its model is shown as

(10) $$\begin{array}{c} E\left(v,h,\theta \right)=-\sum_{i=1}^{m}\sum_{j=1}^{n}v_{i}h_{i}\omega_{ij}-\sum_{i=1}^{m}d_{i}v_{i}-\sum_{i=1}^{m}c_{i}h_{i}, \end{array}$$

where *v*_*i*_ and *c*_*i*_ represent the bias of the *i*-th display layer and its corresponding display neuron, respectively; *ω*_*ij*_ represents the weight value of neuron connection between the display layer and the hidden layer; *h*_*i*_ represents the *j*-th hidden layer; corresponding *d*_*i*_ represents the paranoid value of neurons in the hidden layer; *θ*={*ω*_*ij*_, *c*_*i*_, *d*_*i*_}.

The calculation method of weight value is

(11) $$\begin{array}{c} \omega_{ij}=\eta \left[E_{\text{data}}\left(v_{i}h_{i}\right)-E_{\mathrm{mod}}\left(v_{i}h_{i}\right)\right], \end{array}$$

where *i* and *j* represent the number of visible layer nodes and the number of hidden layer nodes, respectively; *η* represents the efficiency value of learning; simultaneous *v*_*i*_ and *h*_*i*_ represent binary variables; *E*_data_ and *E*_mod_ represent the expected value of the training sample and the output sample, respectively.

## 3. Feature Learning Algorithm Based on CNN&DBN to Extract Music Score

With the development of Internet and digital audio technology, music information retrieval has gradually become a research hotspot. The effective recognition and classification of music genres are an important research direction. It is also a hot research direction to identify the type of musical instrument and determine the specific name of musical instrument in the unknown music segment [20]. The correct extraction of musical features is an important indicator for the classification of musical factions. The vast majority of music songs contain the corresponding musical instrument audio, so the effective recognition and classification of musical instruments can provide strong support for music information retrieval. Therefore, the recognition and classification of musical instruments are also an important part of music information retrieval, which is of great significance to music retrieval. For people, the recognition and classification of musical instruments are relatively easy to complete, as long as the person has a strong musical accomplishment; it can be more accurate recognition and classification of musical instruments, but most of the ordinary music audience does not have this ability, so it is necessary to teach the computer how to automatically identify and classify musical instruments. From an acoustic point of view, the timbre of an instrument is the main basis for distinguishing one instrument from another, accurately marking the unique characteristics of each instrument [21–23]. The timbre of a musical instrument is mainly determined by the vibration of the articulating part of the instrument. Different vibration states lead to different overtones and waveforms, and the proportion of harmonics in overtones determines the timbre of musical instruments. However, the proportion of overtones varies widely, and the different playing techniques used for the same instrument can also show significant changes in timbre, which can also be mistaken for the sound produced by other instruments. This will also lead to difficulties in the feature extraction of musical instruments from musical signals, which will lead to low accuracy in the recognition and classification of musical instruments. There are few research works on musical instrument recognition and classification.

According to the background of the continuum hypothesis and its analogy to the sound signal, it can be seen that the musical information is composed of a large number of sampling points, and there are gaps in time between the sampling points. Therefore, the musical information composed of discrete sampling points can be regarded as composed of numerous frames distributed continuously without gaps. Since the speech signal is a nonstationary signal, it is meaningless to directly extract all the features of a complete audio file, but the audio changes slowly in a short time and can be considered short-term stationary. At this point, the assumption of stability continuity allows us to estimate statistical information from observational data. This short stationary partition is the signal frame. On the whole, the characteristic parameters of music signal change with time, so it belongs to a nonstationary state and cannot be analyzed by the relevant techniques for processing stationary signals. However, considering that the voice in the song is formed by the movement of people's oral muscles, this kind of oral muscle movement is relatively slow and has a lag. At the same time, the accompaniment in the song is formed by the process of percussion and string vibration of piano, guitar, and other instruments, so it also has a certain lag. From this point of view, although the music signal has the time-varying characteristics mentioned above, it can still be regarded as a quasi-static process by extracting a certain short-time interval of the signal; that is, it shows that the music signal has short-time stability.

**Figure 7:**
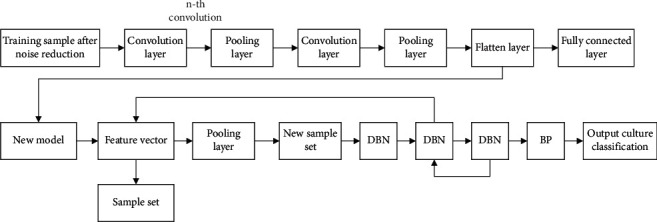
Structure diagram of feature learning algorithm of CNN&DBN.

The advantages of DBN model are that it overcomes the problems of neural network algorithm, such as high requirement on data, very slow convergence rate, and poor global optimization of local solution. Combined with the advantages of learning algorithm, this paper proposes CNN & DBN algorithm, which uses the characteristics of learning algorithm to preprocess and extract features of each musical instrument music sample score [24]. Then it is input into the deep confidence network, followed by adjusting the parameter to predict the types of test instruments, and, through repeated comparison test and tuning, the highest recognition accuracy is finally reached. The structure diagram of feature learning algorithm of CNN&DBN is shown in Figure 7.

The detailed steps are as follows:

1. Firstly, the input dataset is preprocessed, mainly through the pitch feature matrix and constant changes to complete noise filtering and volume calibration. After preprocessing, the sample dataset of music soundtrack is obtained.
2. The improved music score training model based on CNN model is adopted to conduct supervisory learning training on samples and generate training feature sample model.
3. The introduction of the original test sample set also requires noise reduction for the test set. After processing, the feature sample model extracted by CNN was input into the test combination. New feature vectors are extracted.
4. The extracted feature vector is combined with the classification set *Y* of the original sample set to generate a new input sample set, and the sample set is input into the DBN model as the input set for training, and the final classification training result is obtained.

**Figure 8:**
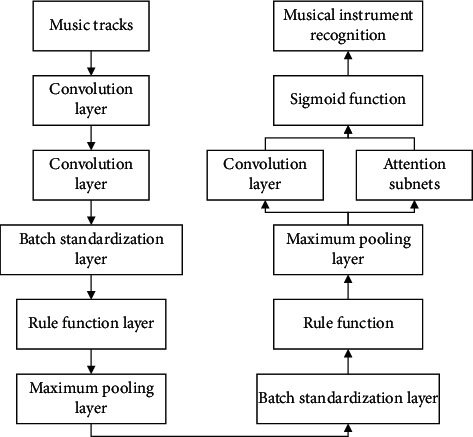
Flow chart of BNN model for music recognition and classification based on focus network.

Based on the research of the new model and the polyphony combination of multiple parts of the score, the algorithm tells CNN to adjust the model algorithm adaptively and introduces the simulated human auditory attention to establish the music score recognition classification benchmark model. The key of the model is the automatic filtering due to the difference of brain structure. When people hear music, they will focus on the rhythm and main sound parts of the music so as to identify different instruments. Therefore, this model modeling method proposed in this paper simulates the operation process of human brain. Combined with the characteristics of background musical instruments, attention model is introduced to set corresponding feature weights for different musical instrument bands corresponding to key components [25]. The flow chart of BNN model for music recognition and classification based on focus network is shown in Figure 8.

As shown in Figure 8, BNN model based on concern network is divided into convolution layer, batch standardization layer, rule function layer, and maximum pooling layer. Among them, the pooling layer is part of attention network and convolution layer, so as to generate feature vectors with weight values, and Sigmoid function is used to obtain recognition feature vectors. The attention network includes the convolution, the whole connection layer and the corresponding normalization function. After the convolution of the characteristic matrix *r*_*n*_, it becomes a medium-sized matrix. Each medium-sized matrix input connection layer will get an appropriate amount of attention *v*_*n*_=*f*_all_(*r*_*n*_ : *θ*_all_), set *θ*_all_ as the parameter of all connection layers, and each component of all attention vectors uses the normalization function to normalize the memory, and the sum *a*_*n*_ exceeds the normalization amount *M* . The attention weight value *∂n* is obtained, as shown in the two following equations:

(12) $$\begin{array}{c} a_{n}=\sum_{m=1}^{M}\frac{\exp\left(v_{n,m}\right)}{\sum_{n=1}^{N}\exp\left(v_{n,m}\right)}, \end{array}$$

(13) $$\begin{array}{c} \partial n=\frac{\exp\left(a_{n}\right)}{\sum_{n=1}^{N}\exp\left(a_{n}\right)},\sum_{n=1}^{N}\partial n=1. \end{array}$$

The flow of BNN model based on concerned network is similar to that of traditional neural network algorithm [25]. The core lies in the attraction of attention factor of convolution layer. The number of operations and space occupation of single-layer convolution in this algorithm are shown in the two following equations:

(14) $$\begin{array}{c} T∼O\left(\sum_{i=1}^{D}M_{l}^{2}\cdot K_{l}^{2}\cdot C_{l-1}\cdot C_{l}\right), \end{array}$$

(15) $$\begin{array}{c} S∼O\left(\sum_{i=1}^{D}K_{l}^{2}\cdot C_{l-1}\cdot C_{l}+\sum_{i=1}^{D}M_{l}^{2}\cdot C_{l}\right), \end{array}$$

where *M* represents the length of the convolution kernel corresponding to the eigenvalue output by the convolution kernel in the convolution layer; *C*_*l*−1_ represents the input channel value corresponding to the upper layer of the convolution kernel; *C*_*l*_ represents the output channel value of this layer; *M*_*l*_^2^ represents the area of the eigenvector; *K*_*l*_^2^ represents the calculated area of the convolution kernel; *D* represents the depth of the network. Thus, the value of the channel is the depth of the convolution. Therefore, the time complexity of the convolution algorithm is obviously positively correlated with the area of the convolution layer and the feature vector [26–28].

## 4. Example Verification and Analysis

**Figure 9:**
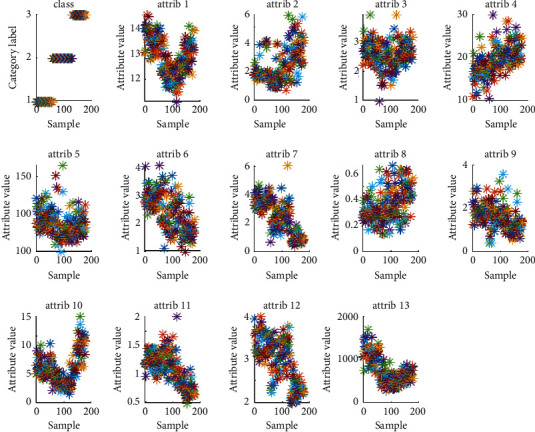
Attribute distribution diagram of feature samples.

The data samples selected in this paper are 3 seconds long and contain music clips of various musical Instruments, while the musical instrument training samples contain 3120 samples of musical instrument, as well as a total of 21,840 music samples. Musical instruments selected 670 test samples, with a total of 4690 test samples. In the pretreatment process, it is preferred to add labels after noise reduction to the samples. The model of the training set is used to cross process the test set and the training set. At the same time, it also verifies the prediction ability of the score of the test samples. Finally, the predicted score labels are compared with the actual score classification. The final accuracy is the result of the average calculated value. The attribute distribution diagram of feature samples is shown in Figure 9.

**Figure 10:**
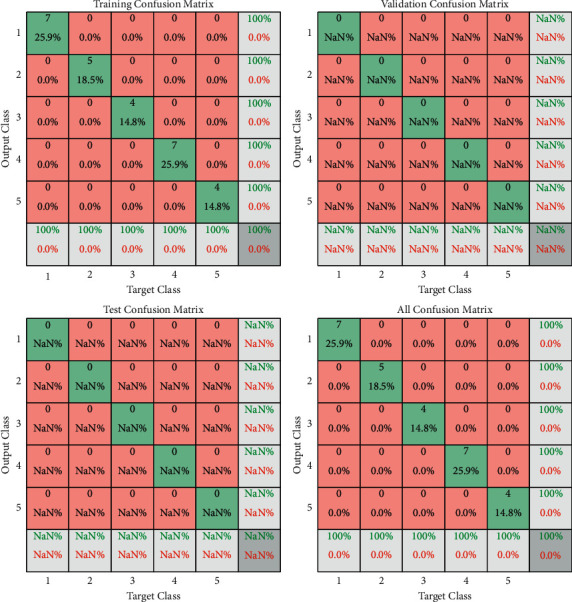
Contrast of confusion matrix.

Combined with the research on the performance of the traditional classification algorithms, it was found that deep learning sample training process requires tremendous force and execution time is longer, and the conventional experimental environment is extremely easy to interrupt the experiment process and system outage; the unit usually needs weeks or even months of comparative experiments to achieve the basic state of stable operation. Other algorithms will also be compared [29]. This paper adopts the finite cycle method to carry out the experiment, limiting the execution cycle and the number of cycles. After repeated calculation, 9 cycles are selected to be executed. The accuracy comparison experiment between the CNN&DBN model tested in this paper and the decision tree is carried out. As well as the change of the execution accuracy, the experimental results show that the performance is best when the number of cycles is 9. The contrast of confusion matrix is shown in Figure 10. Compared with the average accuracies of other classical algorithms, the algorithm in this paper combines the advantages of CNN in feature extraction and the high execution efficiency of DBN algorithm and introduces attention weight into CNN algorithm. Therefore, both accuracy and performance are greatly improved, which are significantly higher than those of other algorithms.

**Figure 11:**
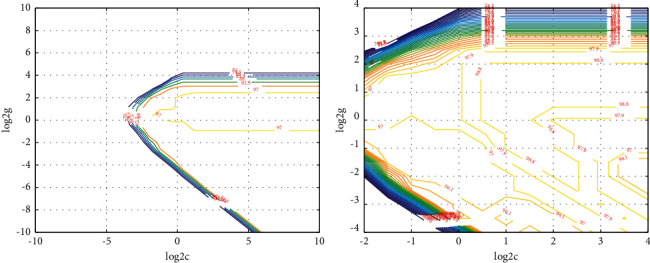
Parameter optimization graph of deep learning network.

The music recognition algorithm based on CNN&DBN proposed in this paper has significantly improved the recognition results applied to the same dataset in terms of various scores and overall recognition accuracy compared with other classical models, especially the recognition improvement effect of violin is more obvious. Compared with other models, the overall music recognition of this model is more uniform, which effectively improves the problem of uneven recognition effect between different categories. By changing the proportion of labeled samples in the training set of traditional Chinese musical instruments for network fine-tuning training [30], the training set is constant, and the recognition accuracy of musical instruments increases with the increase of the number of labeled samples in network fine-tuning. However, the accuracy of the algorithm can still reach more than 90%. In this way, when a large number of Chinese traditional musical instrument music samples are identified and classified by deep confidence network, only a small number of samples need to be labeled, which greatly reduces the workload and is conducive to improving the efficiency of identification and classification. The parameter optimization graph of deep learning network is shown in Figure 11.

**Figure 12:**
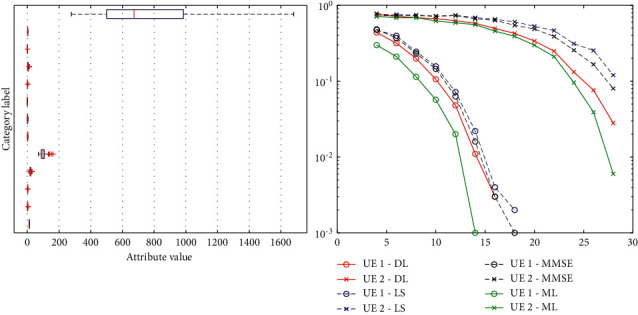
Characteristic curves for different instruments.

By comparing the results in the figure, we find that the first hidden layer has the greatest influence on the predicted results, and when the number of nodes in the first hidden layer is about 3 times of the characteristic dimension of the input sample, the performance of the network converges basically [31]. Meanwhile, as the number of neuron nodes in each layer increases, the training duration will also increase. Therefore, except for the first layer, the number of neuron nodes in other hidden layers will be less appropriately selected. Taking the 2s-long Chinese traditional musical instrument music fragment as a sample, the training set contains 2340 samples for each type of musical instrument, with a total of 14,040 musical instrument samples. The validation set and test aet contain 780 musical instrument samples per category, for a total of 4,680 musical instrument samples. First, all musical instrument samples were labeled, and the training set was used to train the model. Then, cross-validation was carried out on the validation set, and the musical instrument samples in the test set were predicted. Finally, the average recognition and classification accuracy was obtained by comparing the predicted musical instrument labels with the actual musical instrument labels. The characteristic curves for different instruments are shown in Figure 12.

**Figure 13:**
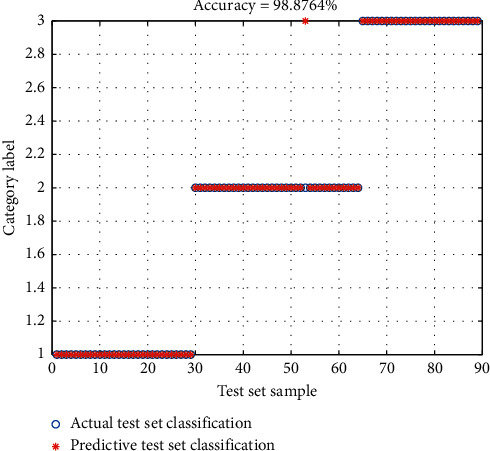
Classification accuracy of the classifier.

The recognition and classification effect of MFCC features directly input into the traditional classical classifier is poor, and the lowest recognition and classification accuracy of decision tree is 83.9%, and the *k*-nearest neighbor classification algorithm (KNN) has the highest average classification accuracy of 92.7%. The accuracy rate is even as high as 99.2%, which is 6.5% higher than that of KNN. The experimental results prove that the music samples of traditional Chinese musical instruments can have better recognition and classification effect after further learning by deep confidence network. By comparing the recognition rate confusion matrices of the six classification methods, it is found that different classification methods have different recognition effects on various traditional musical instruments, which may be related to the training of the inherent characteristics of musical instruments in these classifiers. In traditional identification method, erhu and flute recognition rate is low, but, with deep belief networks of instrument samples after further training, the two kinds of musical instrument recognition rate also have the obvious improvement, which proves the recognition and classification task of deep belief network for traditional instruments. The advantage of using this method is that more musical features of the essence of traditional instruments can be extracted. The classification accuracy of the classifier is shown in Figure 13.  Table 1 shows comparison between algorithms for each score recognition result.

## 5. Conclusion

With the continuous development of machine learning, more and more practical application problems have been put forward, among which the recognition of music score is still a challenge. In this paper, convolutional neural network (CNN) is improved to identify and extract feature vectors of music soundtrack, and the high efficiency of the implementation of deep confidence network (DBN) is introduced. Taking the feature vector set extracted by CNN as DBN input set, a feature learning algorithm based on CNN-DBN was established to extract music score. Through the comparison of experiments, it is found that the model proposed in this paper shows more accurate recognition ability and good execution performance in the recognition ability of multiple types of polyphony music. The recognition rate of the improved algorithm applied to music recognition is as high as 98.4%, which is obviously better than those of other classical algorithms. The experimental data fully demonstrate the obvious advantages of the proposed algorithm in the recognition of music soundtrack and further prove the superiority of using the deep confidence network to identify and classify musical instruments.

Although the research in this paper has achieved some results compared with the traditional methods, due to the limitations of my theoretical level and research time, there are still many deficiencies in this paper which need to be improved. The following aspects need to be improved in the follow-up research work.

Firstly, in the Internet information resources, the information base of audio retrieval is very small. How to make the retrieval system adapt to the massive music information retrieval needs further study.

Secondly, the database capacity of the two experiments in this paper is relatively small, which only contains tens of thousands of music samples. In the face of massive music data on the network, the computational complexity of training is very high, and the hardware may not be able to afford it. We should try to learn how to do parallel processing and how to implement it with GPU, which is also a direction of future research.

Third, in the experiments based on HMM and CDBN model, the retrieval recognition rate for melody is not good enough, which is difficult to make many people able to better obtain the music information they need, so in the future music retrieval system in this aspect of the related technology needs to make breakthroughs.

Fourth, convolutional deep confidence network is time-consuming in training, and there is no unified theoretical guidance for network structural parameters. Therefore, how to set network structural parameters adaptively according to different samples is worth discussing.

## Figures and Tables

**Table 1:** Comparison between algorithms for each score recognition result.

Method	Piano	Violin	Viola	Bass	Saxophone	Xylophone
Decision tree	0.899	0.901	0.892	0.879	0.832	0.836
KNN	0.891	0.894	0.835	0.887	0.875	0.841
SVM	0.902	0.876	0.894	0.908	0.894	0.855
CNN&DBN	0.913	0.945	0.931	0.903	0.903	0.863

## Data Availability

The dataset can be accessed upon request.
